# Transcriptomic Analysis of *Staphylococcus xylosus* in Solid Dairy Matrix Reveals an Aerobic Lifestyle Adapted to Rind

**DOI:** 10.3390/microorganisms8111807

**Published:** 2020-11-17

**Authors:** Sabine Leroy, Sergine Even, Pierre Micheau, Anne de La Foye, Valérie Laroute, Yves Le Loir, Régine Talon

**Affiliations:** 1 Université Clermont Auvergne, INRAE, MEDIS, Clermont-Ferrand, France; pierre.micheau@inrae.fr (P.M.); regine.talon@inrae.fr (R.T.); 2 INRAE, Agrocampus Ouest, STLO, Rennes, France; sergine.even@inrae.fr (S.E.); yves.le-loir@inrae.fr (Y.L.L.); 3 INRAE, UMRH, Saint-Genès-Champanelle, France; anne.delafoye@inrae.fr; 4 TBI, Université de Toulouse, CNRS, INRAE, Toulouse, France; valerie.laroute@insa-toulouse.fr

**Keywords:** *Staphylococcus*, cheese model, physiology, nutrient shortage, osmotic stress, oxidative stress

## Abstract

*Staphylococcus xylosus* is found in the microbiota of traditional cheeses, particularly in the rind of soft smeared cheeses. Despite its frequency, the molecular mechanisms allowing the growth and adaptation of *S. xylosus* in dairy products are still poorly understood. A transcriptomic approach was used to determine how the gene expression profile is modified during the fermentation step in a solid dairy matrix. *S. xylosus* developed an aerobic metabolism perfectly suited to the cheese rind. It overexpressed genes involved in the aerobic catabolism of two carbon sources in the dairy matrix, lactose and citrate. Interestingly, *S. xylosus* must cope with nutritional shortage such as amino acids, peptides, and nucleotides, consequently, an extensive up-regulation of genes involved in their biosynthesis was observed. As expected, the gene *sigB* was overexpressed in relation with general stress and entry into the stationary phase and several genes under its regulation, such as those involved in transport of anions, cations and in pigmentation were up-regulated. Up-regulation of genes encoding antioxidant enzymes and glycine betaine transport and synthesis systems showed that *S. xylosus* has to cope with oxidative and osmotic stresses. *S. xylosus* expressed an original system potentially involved in iron acquisition from lactoferrin.

## 1. Introduction

Cheeses are one of the oldest fermented products, representing more than 1400 varieties of traditional cheeses around the world and nearly 1000 varieties in France [1]. Their microbial communities, composed of “house microbiota” and starters, are shaped by the technological processes and contribute to the development of the sensorial properties of cheeses. Studies of this microbial diversity combining phenotypic and genomic approaches have revealed two ecosystems, the core and the rind. Lactic acid bacteria (LAB) largely dominate the inner part of the cheese [1,2,3]. The study of rinds from 137 different cheeses from 10 countries and 12 different French cheeses revealed a dominant core of 14 bacterial and 10 fungal genera [1,4]. The genus *Staphylococcus* is one of the genera of the bacterial core in milk and cheese [1,4,5,6] and is often identified in different types of cheese [7,8,9,10]. Within this genus, *Staphylococcus xylosus* has been identified in soft red smeared cheeses, Raclette, Saint Nectaire [2], and Livarot [11]. *S. xylosus* was enumerated at 10^5^ to 10^9^ CFU/g in Camembert and blue-veined cheeses [12]. *S. xylosus* is also commercially available as an adjunct culture to enhance the aroma and texture of cheeses, as well as the color of the surface of smeared cheeses [13].

The transcriptomic or more recently the metatranscriptomic responses of LAB have been well established in milk and cheese [14,15,16,17,18,19]. The potential of surface microbiota has been less investigated. Several studies have characterized the role of yeasts, in co-culture with LAB, and/or in association with other ripening bacteria [20,21,22,23]. Very few studies have considered coagulase-negative staphylococci, despite their frequency in cheese. The investigation of interaction between *S. xylosus, Lactococcus lactis*, and *Yarrowia lipolytica* in culture media revealed that the expression of some genes of *S. xylosus* such as the lactate dehydrogenase gene (*ldh*) and genes involved in amino acid transport, significantly decreased in mixed cultures [24]. Three studies have characterized the transcriptomic response in milk or cheese of *Staphylococcus aureus* in mixed cultures with different species of lactic acid bacteria, revealing in particular the increased expression of genes associated with the acid and redox stress response, the repression of certain enterotoxin genes and of the *agr* system, a major virulence regulator [25,26,27].

The adaptation mechanisms of *S. xylosus* in cheese are poorly documented despite its importance for cheese flavor development and typicity. To better understand *S. xylosus* behavior in a dairy matrix at the molecular level, we analyzed the transcriptome of *S. xylosus* in a solid dairy matrix incubated for 48 h in conditions that mimic the fermentation step. The in situ response of *S. xylosus* was analyzed at 24 and 48 h vs 6 h.

## 2. Materials and Methods

### 2.1. Dairy Matrix and Inoculation

The dairy matrix was made as described previously [28]. In brief, retentate was prepared from 5.5-fold concentrated ultra-filtered cow’s milk supplemented with salt (0.6%) and UHT cream (5.2%). Its composition was: dry matter, 258.5 g/kg; fat, 52.3 g/kg; lactose, 40.9 g/kg; total nitrogen, 140.15 g/kg; noncaseinic nitrogen, 26.9 g/kg; nonprotein nitrogen, 1.61 g/kg; and NaCl, 6.2 g/kg. The pH was 6.54.

The *S. xylosus* C2a strain, whose complete genome is available (LN554884), was used in this study. It was grown for 15 h at 30 °C with shaking (150 rpm) in brain-heart infusion (BHI) broth (Becton, Dickinson and Compagny, Le Pont de Claix, France). Then, the culture was centrifuged and inoculated at a concentration equivalent to 10^6^ CFU/mL in the retentate heated at 25 °C before addition of 0.3 µL/mL of rennet (DSM Food Specialties; Delft, Netherlands). The dairy matrix (per 30 g) was incubated for 6 h at 30 °C and then the solid dairy matrix was transferred at 12 °C into an incubation chamber. Three independent experiments were done.

The bacterial cells were enumerated immediately following inoculation and after 6 h, 24 h, and 48 h of incubation. The numbers of CFU were determined after serial dilutions on plates of BHI agar incubated at 30 °C for 24 h.

At 6, 24, and 48 h of incubation, 200 mg samples were taken and immediately frozen in liquid nitrogen to stabilize the bacterial RNA.

### 2.2. RNA Extraction and Purification

RNA was extracted from the bacterial cells separated from the solid dairy matrix according to the protocol described previously [28]. Briefly, the samples were thawed and homogenized in trisodium citrate solution (2% *w/v*) at 4 °C using a mechanical Waring blender. The cells were then recovered by centrifugation for 5 min at 6000× *g* at 4 °C and washed with TE buffer (10 mM Tris-HCl, 1 mM EDTA, pH 8.0). the cell pellets were resuspended in Tris-EDTA buffer (20 mM Tris-HCl, 2 mM EDTA, pH 8.0) and RLT buffer (RNeasy Mini Kit, Qiagen, Courtaboeuf, France) at a ratio of 30/70 (*v/v*). After addition of chloroform, the samples were vigorously shaken in a bead beater (Biospec Products Inc., Bartlesville, OK, USA) and centrifuged for 20 min at 12,000× *g* at 4 °C. The RNA was isolated from the aqueous phase using RNeasy Mini Kit according to the manufacturer’s instructions. DNase treatment of RNA was performed with Turbo DNAse (Ambion, Austin, TX, USA) according to the manufacturer’s instructions. The absence of *S. xylosus* genomic DNA contamination was verified by PCR. DNase treatment of RNA was performed with Turbo DNAse (Ambion, Austin, TX, USA) according to the manufacturer’s instructions. The absence of *S. xylosus* genomic DNA contamination was verified by PCR. Total RNA isolated was quantified using a Nanodrop 1000 (Thermo Fisher Scientific, Wilmington, DE, USA) and RNA quality was analyzed using an Agilent 2100 Bioanalyzer (Agilent Technologies, Santa Clara, CA, USA) according to the manufacturer’s instructions. The RNA was stored at −80 °C.

### 2.3. RNA Labeling and Microarray Analyses and Validation

The RNA labeling and hybridization on the Agilent microarray were carried out as described previously [29]. A complete description of the array developed for *S. xylosus* C2a is available at the NCBI Gene Expression Omnibus (GEO) database under platform accession number GPL19201. The microarrays were analyzed as described previously [29]. Significant differences in the probe set intensities between the conditions were identified using a linear model with an empirical Bayes method using all information probes to moderate the standard errors of the estimated log-fold changes [30]. The probabilities were corrected by the Benjamini–Hochberg procedure in order to control the false-discovery rate (FDR) with a *p* value cut-off of 0.05. All the probes with an FDR ≤ 0.05 were considered to be differentially expressed. Finally, a gene was considered to be differentially expressed if at least 50% of the corresponding probes were differentially expressed and if the ratio of expression was ≥2 or ≤0.5.

The microarray data were validated as described previously [29]. The targeted genes for qPCR and primer sequences are listed in Supplementary Table S1. The analyses were performed on the same samples of RNA as used for the microarray experiments. The relative fold change of gene expression, using measured *tuf* housekeeping gene expression, was determined by the 2^−ΔΔCt^ method [31].

### 2.4. Chemical Analysis of the Solid Dairy Matrix

The methods for determining sugar, organic acid, and free amino-acid content were carried out as described previously [14].

## 3. Results and Discussion

### 3.1. Growth of S. xylosus in the Solid Dairy Matrix and Transcriptome Profile

*S. xylosus* was inoculated in the milk retentate at 5.6 log CFU/g. The dairy matrix was immediately solidified by addition of rennet. *S. xylosus* grew and reached 7.4 log CFU/g after 6 h of incubation at 30 °C. At 6h-post inoculation, the temperature of incubation was reduced to 12 °C, the strain went up growing with a reduced slope and reached 8.7 log CFU/g, 24 h post-inoculation. Then, the population remained almost at this level (8.9 log CFU/g) until the end of the experiment (48 h). The initial pH was 6.54 and did not vary during the incubation. Similarly, *Staphylococcus aureus* grown in the same solid dairy matrix did not acidify the medium [25].

The in situ *S. xylosus* response revealed a change in gene expression at 24 and 48 h of incubation in the solid dairy matrix in comparison with the 6 h time point of sampling used as reference in our study. There were 1175 genes differentially expressed at 24 and 48 h, with 779 genes in common (421 up-, 358 down-regulated), indicating that transcriptional changes had occurred at 24 h and lasted up to 48 h (Supplementary Table S2). These differentially expressed genes were classified into different functional categories: the most represented being metabolism (29%), information storage and processing (14%), and cellular processes (9%).

To validate the microarray analysis independently, the relative expression of 62 differentially expressed genes representing about 8% of the genes at both 24 and 48 h was measured by qPCR (Supplementary Table S1). The microarray and qPCR results for the tested genes were correlated for the two times of incubation (24 h: r² = 0.911, slope y = 1.1178x; 48 h: r² = 0.928, slope y = 1.3302x) and the expected trend in the expression pattern was confirmed.

The transcriptomic profile illustrated a general slowdown of *S. xylosus* activity at 24 and 48 h. Not only were the genes encoding the translation machinery, synthesis, and modifications of ribosomal proteins (14 genes *rps*, 14 genes *rpl*, 6 genes *rpm*) underexpressed but so were the genes involved in DNA replication, recombination, and repair (Supplementary Table S2). This underexpression of ribosomal proteins attested to the decrease in growth rate of *S. xylosus* observed by cultural approach. Such down-regulation of genes involved in replication and translation machineries has already been observed for *Lactococcus lactis* cultured in the same cheese model after 24 h of growth in the same conditions [14]. This decrease in *S. xylosus* growth was accompanied by the induction of the *rnr* gene involved in ribosome degradation and *uvrAB* genes in DNA excision repair (Table 1). Genes involved in cell division, peptidoglycan synthesis (*divIB*, *murD*, *mraY*, *pbp1*, *ftsL*), and cell lysis (phage) were underexpressed for *S. xylosus* grown in the dairy matrix (Supplementary Table S2), while all these genes were overexpressed in *S. xylosus* grown in a meat model [32]. This could be due to the decrease in temperature (from 30 to 12 °C) applied to the dairy matrix to mimic cheese making process. However, the transcriptomic data did not reveal any induction of genes involved in adaptation to cold, e.g., *cspA* encoding a cold shock protein was underexpressed (Table 1). In relation with this slowdown activity, only the cluster *hslUV*, a member of the Hsp100 (Clp) family of ATPases, was overexpressed (Table 1). This ATP protease is necessary for cellular protein homeostasis and protein quality control [33].

### 3.2. Aerobic Carbohydrate Catabolism

**Figure 1:**
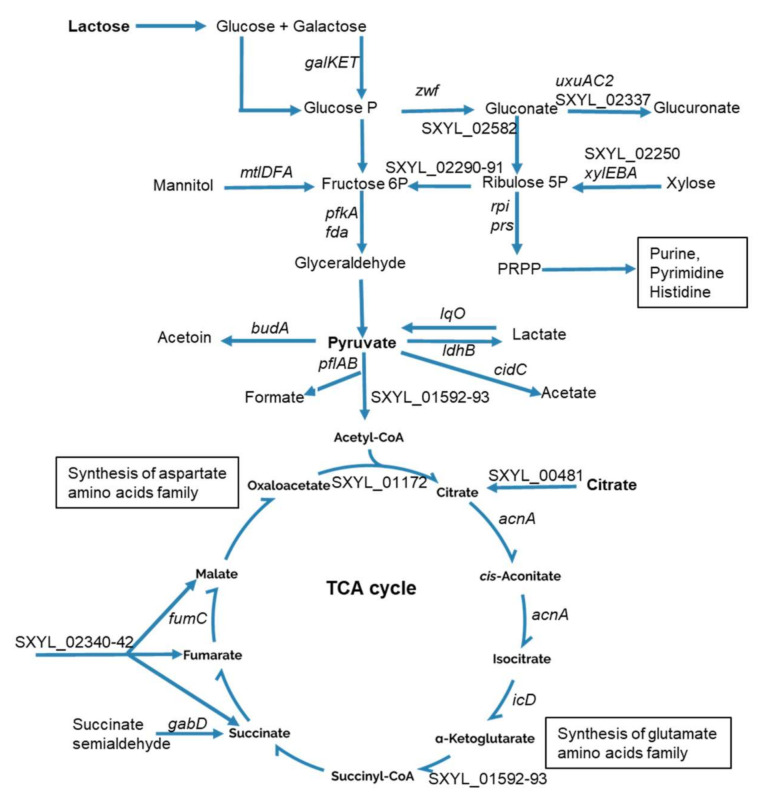
Summary of carbohydrate catabolism by *Staphylococcus xylosus* in solid dairy matrix showing the overexpressed genes (the level of expression of these genes and the name of the corresponding enzymes are given Table 1).

The main carbohydrate in the dairy matrix is lactose. Its initial concentration was 113.4 mM and it slightly decreased during incubation. Only 7.5 mM of lactose was consumed by *S. xylosus* after 48 h of incubation; lactate and acetate were not detected, and pH did not vary. Interestingly, similar results were observed for *S. aureus* grown in milk or in cheese, with very low or no consumption of lactose and no detection of lactate and acetate [25,27]. Like *S. aureus*, *S. xylosus,* however, has the genetic potential to assimilate lactose via a system composed of two genes, *lacP* and *lacH*, which, respectively, encode a lactose permease and a β-galactosidase, which hydrolyzes lactose to glucose and galactose [34,35]. A *lacR* regulatory gene is positioned upstream of the operon and oriented opposite to the *lacPH* operon [34]. In our conditions, *lacR* was underexpressed (Table 1), which might have resulted in low and constitutive *lacPH* expression in *S. xylosus* as previously observed [34]. The *lacPH* expression was also subject to carbon catabolite repression mediated by CcpA encoded by *ccpA* overexpressed in our study at 48 h (Table 1) which might explain the low expression of *lacPH* in our conditions. Apart from lactose related genes, we found several genes encoding phosphoenolpyruvate-dependent sugar phosphotransferase systems (PTS) that were highly overexpressed with the cluster SXYL_00773-76 coding for the mannitol transport system and another uncharacterized cluster SXYL_00255-260 (Table 1). The operon *galKET* was overexpressed at 24 h of incubation. It encodes enzymes involved in the degradation of galactose to glucose 1-P. Then, in our conditions, glucose 1-P could be further metabolized *via* the pentose phosphate (PP) and pentose/glucoronate interconversion pathways as shown by 15 genes overexpressed in these pathways (Table 1, Figure 1).

Two genes (*fda, pfkA*) of the Embden–Meyerhof–Parnas (EMP) pathway related to fructose catabolism were overexpressed, as was the cluster *mtl*, which could lead to fructose. The glycolytic operon regulator *gapR* was up-regulated. In *S. aureus*, *gapR* down regulates the glycolytic operon (*gap*, *pgk*, *tpi*, *pgm*, *eno*) [36], which was underexpressed in our conditions (Supplementary Table S2).

Pyruvate is a nexus between several metabolic pathways, and it can be catabolized into several metabolites (Figure 1). These include catabolism into lactate by lactate dehydrogenase encoded by *ldhB* in *S. xylosus*. Here, we found *ldhB* to be overexpressed, as was *lqo*, which encodes the reverse reaction (Table 1, Figure 1), this bidirectional flow could explain we did not measure lactate. Pyruvate can also lead to acetate, the cluster *cidBC* being highly overexpressed in the dairy matrix. The *cidC* gene encodes a pyruvate oxidase, which converts the excess of pyruvate into acetate. Interestingly, this enzyme has been shown to be coupled to aerobic respiration due to its ability to shuttle electrons to quinone intermediates [37]. The acetate can then be oxidized in the TCA cycle to generate energy [38]; this could explain why we did not measure acetate under our conditions. Pyruvate can also be catabolized into formate and acetyl CoA and the *pflAB* operon encoding the pyruvate formate lyase was overexpressed at 24 h. Likewise, pyruvate formate lyase was shown to be the major up-regulated protein in *Streptococcus thermophilus* grown in milk [39]. Pyruvate can also be catabolized to acetyl CoA by 2-oxoglutarate ferredoxin oxidoreductase encoded by SXYL_01592-93 overexpressed. In both cases, acetyl-CoA fuels the TCA cycle. Finally, pyruvate can lead to the synthesis of acetoin, a compound involved in butter aroma, by the acetolactate decarboxylase encoded by *budA* overexpressed in *S. xylosus* (Table 1, Figure 1).

Citrate was assayed in the dairy matrix. Its initial concentration was about 3 mM, and 0.46 mM was consumed by *S. xylosus* after 48 h of incubation. In line with these observations, the gene SXYL_00481 encoding a citrate transporter was overexpressed (Table 1, Figure 1). Furthermore, the cluster SXYL_02340-42 encoding a transport system for C4-dicarboxylates such as malate, fumarate, and succinate was also overexpressed. All these transporters can feed the TCA cycle at one step or another. Eight genes encoding enzymes of the TCA cycle were found overexpressed in this study (Table 1, Figure 1). These genes encode enzymes leading to the production of α-ketoglutarate and oxaloacetate involved in amino acid synthesis. Consistent with what we observed in *S. xylosus*, *L. lactis* grown in skimmed milk or in a cheese model consumes citrate, with concomitant positive regulation of the *cit* operon [14,40]. However, consumption of citrate by *L. lactis* was complete (from 12 mmol.kg^−1^ to 0) after 24 h of incubation whereas it was rather slow or incomplete in *S. xylosus* with only 15% of citrate consumed after 48 h. 

Finally, the operon *cydBA* and the gene *ppk* involved in oxidative phosphorylation and SXYL_01851 encoding a glutaredoxin functioning as electron carriers were up-regulated (Table 1) and could contribute to the energy used to produce adenosine triphosphate (ATP).

These data, in particular the up-regulation of the genes of the TCA cycle together with the absence of acidification corroborated by absence of lactate and acetate suggested that *S. xylosus* adopted an aerobic lifestyle in the dairy matrix.

### 3.3. Amino Acid Synthesis

The dairy matrix was rich in protein essentially casein but deficient in free amino acids, their concentration being below the detection limit of 25 µM. As a result of these amino acid deficiencies, only four genes encoding amino acid transport systems were overexpressed by *S. xylosus* (Table 1). A gene potentially involved in peptide transport, *ctsA*, was also overexpressed (Table 1). In *Escherichia coli* and *Campylobacter jejuni,* CstA was shown to be involved in peptide transport and *cstA* was overexpressed when cells entered the stationary phase [41,42].

**Figure 2:**
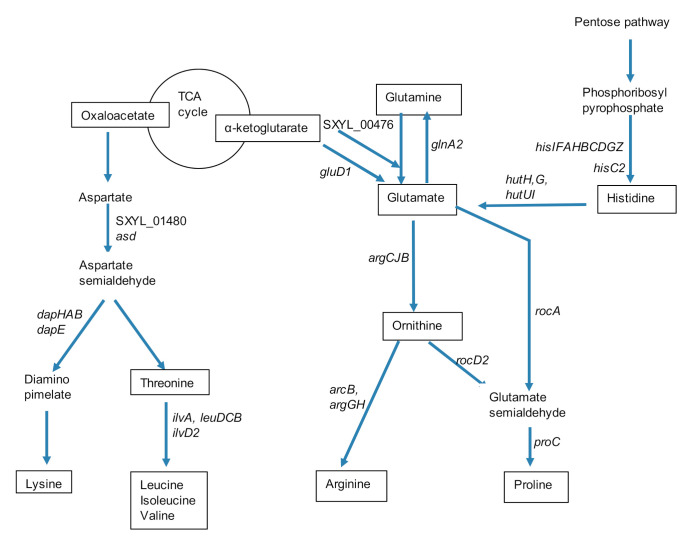
Summary of synthesis pathways for amino acids of the glutamate and aspartate families and for histidine by *Staphylococcus xylosus* in solid dairy matrix showing the overexpressed genes (the level of expression of these genes and the name of the corresponding enzymes are given Table 1).

*L. lactis* had an efficient proteolytic system to cope with this low concentration of free amino acids [14]. *S. xylosus* did not hydrolyze casein, but it is prototrophic and can grow on a medium containing ammonium as the sole nitrogen source [43]. In support of this, bioinformatic analyses of the *S. xylosus* genome revealed a complete repertoire of biosynthetic operons needed to synthesize all 20 amino acids [35]. Consequently, *S. xylosus* overexpressed genes involved in several pathways of amino acids synthesis in the dairy matrix (Table 1). Intriguingly, the transcriptional pleiotropic repressor *codY* was also up-regulated. In *S. aureus*, Cod Y acts as a direct repressor of transcription of genes encoding amino acid biosynthesis, and amino acid and peptide transport, but carbon or nitrogen limitation relieved CodY regulation [44]. Thus, in our conditions, *S. xylosus* must have perceived a nitrogen limitation that relieved codY repression. Consistent with that, a cluster of 9 *his* genes and the *hisC2* gene involved in histidine synthesis were overexpressed (Table 1, Figure 2). Then, histidine can be degraded by a four-step pathway to glutamate, the 4 *hut* genes encoding these enzymes being overexpressed (Table 1, Figure 2). Glutamate can also be synthesized from α-ketoglutarate by a glutamate synthase (encoded by SXYL_00476) or a glutamate dehydrogenase (encoded by *gluD1*). Glutamate can be further catabolized in different pathways. It can lead to glutamine via glutamine synthetase encoded by *glnA2,* which was highly overexpressed; then, glutamine can fuel different pathways such as purine and pyrimidine syntheses. Glutamate can also be catabolized to glutamate 5-semialdehyde and then contributed to proline synthesis (Figure 2). Arginine and proline can be synthesized *via* the glutamate pathway as described for *S. aureus* [45] and illustrated in Figure 2 for *S. xylosus*. Finally, arginine through arginase (*arg*) released ornithine and urea further catabolized in NH3 by urease encoded by a cluster of 6 genes (*ureGFECBA*) overexpressed in our conditions (Table 1). Furthermore *S. xylosus* can import urea (SXYL_00297) suggesting that it can consume urea present in the dairy matrix as it has been already shown for *S. aureus* in a similar matrix [25]. This ammonia will serve as nitrogen source for *S. xylosus* growth. It has been shown that urease is induced in the case of nitrogen starvation in *Bacillus subtilis* and *Corynebacterium glutamicum* [46,47].

The pathway of lysine and branched-chain amino acids synthesis from aspartate is shown in Figure 2. Six genes encoding enzymes involved in the synthesis of lysine were overexpressed. The first two, *asd* and SXYL_01480, were involved in the catabolism of aspartate to aspartate semialdehyde, and the four others were involved in the formation of diaminopimelate, which will further lead to lysine (Table 1, Figure 2). A cluster of 4 genes (*ilvA, leuDCB*) and the gene *ilvD2* encoded enzymes involved in valine, leucine, and isoleucine formation from threonine, were all up-regulated (Table 1, Figure 2). 

Glycine is produced through the interconversion of serine and glycine by L-serine hydroxymethyltransferase providing 5,10-methylene-THF [48]. In our conditions, a cluster of three genes (*gcvTPAPB*) involved in the catabolism of this metabolite was up-regulated leading to the synthesis of lipoylproteins (Table 1).

The synthesis of aromatic amino acids depends on chorismate as common precursor [49]. From this precursor, the tryptophan synthesis involves seven enzymes encoded by the cluster *trpABFCDGE*, which was up-regulated in our conditions (Table 1).

**Figure 3:**
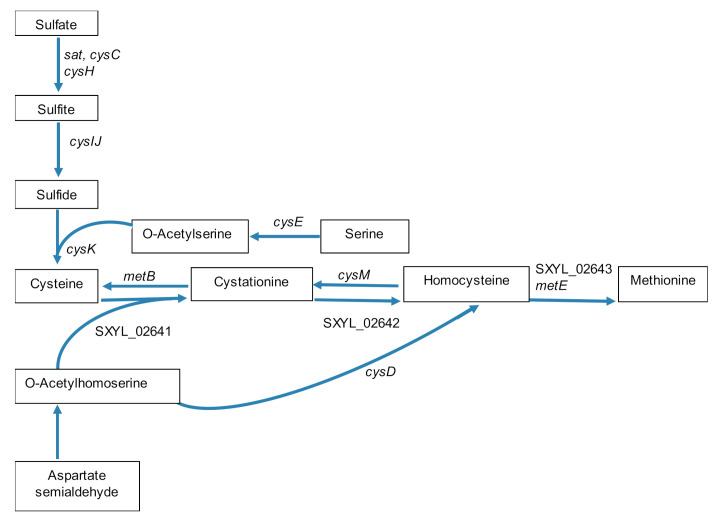
Scheme of synthesis pathways of cysteine and methionine by *Staphylococcus xylosus* in solid dairy matrix showing the overexpressed genes (the level of expression of these genes and the name of the corresponding enzymes are given Table 1).

The synthesis of cysteine and methionine required a thiol group that can be furnished via the assimilatory sulfate reduction pathway as a cluster of nine genes involved in this pathway was strongly up-regulated (SXYL_02630-38, about 40-fold) (Table 1, Figure 3). This cluster presented high similarity with the one described in *B. subtilis* [48]. The sulfide will serve as a thiol group to replace the hydroxyl group of serine for the synthesis of cysteine; this synthesis occurred in two steps in *S. xylosus* encoded by *cysE* and *cysK* (Table 1, Figure 3). The synthesis of methionine could derive from O-acetylhomoserine according to two pathways in *S. xylosus,* as illustrated in Figure 3. In the transsulfuration pathway, O-acetylhomoserine and cysteine can lead to homocysteine and then to methionine by enzymes encoded by genes highly up-regulated (SXYL_02641-42, about 20-fold, SXYL_02643, 60-fold, *metE*, 120-fold) (Table 1, Figure 3). In the alternate pathway, sulfhydration of O-acetylhomoserine to homocysteine occurred via the enzyme encoded by *cysD*, up-regulated 4-fold. It is noteworthy that, the genes *cysM* and *metB* encoding enzymes involved in the formation of cysteine from homocysteine were also overexpressed, but at a level lower than the reverse reaction (Table 1, Figure 3). 

### 3.4. Nucleic Acid Base, Vitamin, and Cofactor Syntheses

Nucleic acid bases are limiting in milk, consequently, *S. xylosus* overexpressed genes encoding enzymes involved in de novo pyrimidine and purine nucleotide biosynthesis. The precursor is the 5-phosphoribosyl-1-pyrophosphate (PRPP) catalyzed by the enzyme encoded by *prs*, which was overexpressed in our conditions (Table 1). The UMP can be synthetized by several pathways in *S. xylosus* [35]. In our conditions, it can be synthetized from: (i) glutamine by enzymes encoded by the operon (*carABpyrBCPR*) overexpressed, (ii) extracellular uracil involving a uracil permease encoded by *pyrP* and a bifunctional protein, encoded by *pyrR,* which has uracil phosphoribosyltransferase activity and regulates expression of the operon, and (iii) the phosphorylation of uridine by a uridine kinase encoded by *udk*, which was overexpressed in our conditions (Table 1).

The pathway for purine first involves the synthesis of IMP in 11 steps as well-described in *B. subtilis* [50]. In *S. xylosus*, the genes required for this synthesis were overexpressed in our conditions (Table 1). Two metabolites of this pathway, the 5-phosphoribosyl-4-carboxamide-5-aminoimidazole and the 5-phosphoribosyl-5-aminomidazole, may be further catabolized to thiamine by enzymes encoded by the overexpressed *thiC, thiD, thiM* genes (Table 1).

Four genes encoding enzymes involved in the synthesis of pantothenate and coenzyme A and three genes involved in nicotinate synthesis were also up-regulated (Table 1). Moreover, the gene *nrlA* encoding an NADPH-dependent oxidoreductase involved in cofactor and vitamin synthesis was highly overexpressed.

### 3.5. Lipid and Glycerolipid Metabolism

The dairy matrix contained fat (52.3 g/kg). *S. xylosus* can catabolize fatty acids in acetyl-CoA by three enzymes, encoded by SXYL_02652-54, which was overexpressed in this environment (Table 1). Acetyl-CoA can also arise from the activity of the enzymes encoded by the cluster SXYL_01253-54, which was overexpressed (Table 1). Acety-CoA will then fuel the TCA cycle. Ten genes encoding enzymes involved in glycerolipid and glycerophospholipid syntheses, which play a key role in membrane biogenesis, were also overexpressed (Table 1).

### 3.6. Iron Homeostasis

Milk and dairy products are considered as poor sources of iron [51]. Iron is associated with casein, citrate, and lactoferrin. Lactoferrin, a member of transferrin family, is a glycoprotein able to bind and transfer iron (Fe^3+^ ions) [52]. Lactoferrin inhibits the growth of iron-dependent bacteria and in certain cases may serve as an iron donor and support the growth of some beneficial bacteria with low iron demands, such as *Lactobacillus* and *Bifidobacterium* species [52,53,54]. Lactoferrin reduces the growth of *S. aureus* and modulates the expression of iron-regulated surface Isd proteins [55]. *S. xylosus* has developed a plethora of mechanisms to acquire iron, including the elaboration of siderophores (*sfa, hts*), the utilization of exogenous siderophores (*sst, fhu*), the acquisition of iron from ferritin (SXYL_00561-63) and the uptake of inorganic free iron (*sit*) [35,56]. In the dairy matrix, the operon SXYL_00561-63 encoding an oxidoreductase, a monooxygenase and a transporter involved in the acquisition of iron from ferritin [56] was highly overexpressed at 24 h (24-fold) and 48 h (36-fold) (Table 1). This assumes that lactoferrin could be the preferred source of iron for *S. xylosus* in the dairy matrix.

### 3.7. Response to Stresses

The *S. aureus* alternative transcription factor SigB is activated by environmental stress and following entry into the stationary phase of cell growth [57]. The *S. aureus* gene *sigB* is in an operon composed of four genes *rsbUVWsigB* [57], and a similar organization was identified in *S. xylosus*. In the conditions used here, three genes of the cluster were found overexpressed in *S. xylosus* (Table 1). The *S. aureus* SigB controls a large regulon of 251 genes, with 198 positively controlled and 53 repressed, including genes involved in cellular processes, intermediary metabolism, and signaling pathways [58]. The *asp23* gene is exclusively regulated by SigB and is a marker of the SigB activity in *S. aureus* [59]. It is the last gene of a cluster of 4 genes, which encodes, in addition to Asp23, an osmoprotectant transporter and two transmembrane proteins, which are well-conserved in staphylococci [60]. These authors [60] suggested a function for Asp23 related to the protection of the cell envelope of non-growing cells. In our study, this cluster (SYL_00743-00746) was also overexpressed (Table 1). Two genes (SXYL_00309, SXYL_00196) encoding proteins involved in universal or general stress were up-regulated. Such general stress proteins (Gsps) were shown to be under the SigB control in *B. subtilis* [61].

Genes encoding proteins involved in transport of cations or anions are positively regulated by SigB in *S. aureus* [57,58]. In *S. xylosus*, the transcription of 8 genes encoding cation transporters and 6 genes encoding phosphate transporters were up-regulated in our conditions (Table 1). In addition to phosphate transporter genes, the genes *ppk*, encoding a polyphosphate kinase, and *ppx*, an exopolyphosphatase, were highly overexpressed (Table 1). These genes could be involved in the synthesis of polyphosphate by using phosphate present in the dairy matrix, either as colloidal particles integrated in the casein micelles or covalently bound to casein molecules as serine-phosphate [62]. Polyphosphates are polymers that contribute to responses to many stresses. In *E. coli,* they are required for stationary-phase survival in response to deficiencies in amino acids or nitrogen [63,64]. The polyphosphates could then contribute to the survival of *S. xylosus* in the dairy matrix deficient in amino acids. 

SigB is also shown to affect pigmentation, with *crtMN* up-regulated in *S. aureus* [58]. In this study, *S. xylosus* overexpressed the cluster *crtOPQM* involved in the carotenoid pigment synthesis pathway and the corollary was that the surface of dairy matrix had an orange color at the end of incubation. This pigment can protect against oxidative stress by scavenging free radicals as it has already been shown for *S. aureus* [65]. *S. xylosus* overexpressed five additional genes in response to this stress (Table 1). Among them, the gene *nos* encoding nitric oxide synthase (NOS) promotes resistance to hydrogen peroxide in *S. xylosus* [66]. These authors demonstrated, that in a *nos* deleted mutant, the expression of genes encoding catalases was modulated, with the up-regulation of *katA* and the down-regulation of *katB* and *katC*. In our study, *katC* was strongly overexpressed, while *katA* was underexpressed (Table 1). In addition, the *sodA* gene encoding superoxide dismutase was overexpressed by *S. xylosus*, as already observed in *S. aureus* for confronting oxidative stress [67,68]. Furthermore, as NO is a highly reactive free radical gas, which at low concentration acts as a signaling molecule and at high concentration promotes nitrosative stress, it can be detoxified by a flavohemoprotein encoded by *hpm* [69], this gene being up-regulated in our conditions (Table 1). Finally, *hslO* was also up-regulated (Table 1). It encoded a redox regulated molecular chaperone, which could protect oxidatively damaged proteins from irreversible aggregation. 

*S. xylosus* had to adapt to NaCl in the dairy matrix. Five genes encoding enzymes involved in transport or synthesis of glycine betaine, a powerful osmoprotectant, were up-regulated (Table 1). The presence of salt also induced three genes encoding sodium:solute symporter and MFS transporters, which could contribute to extrusion of Na [70,71]. The *cap* locus was up-regulated in *S. xylosus* grown in the salted dairy matrix (Table 1). This locus encodes enzymes involved in the synthesis of poly-γ-DL-glutamic acid (PGA) in *Staphylococcus epidermidis,* this polymer protected *S. epidermidis* against a high salt concentration, a key feature of its natural environment, the human skin [72]. Three genes (*isaA, sceD2, sceD1*) encoding transglycolases were overexpressed in *S. xylosus* grown in the dairy matrix. In *S. aureus*, the two proteins, IsaA and SceD, have an autolytic activity and the potential to affect cell separation. The expression of *sceD* is greatly up-regulated by the presence of NaCl [73]. The expression of *sceD* is also positively regulated by SigB, but the greatest effect was that of SaeR, a negative regulator [73]. It is noteworthy that, in our study, the cluster *saeRS* was down-regulated. In *S. aureus*, *isaA* inactivation resulted not only in up-regulation of *sceD* but also of *ssaA*, which encodes staphylococcal secretory antigen, which has peptidoglycan hydrolase activity [73]. The gene SXYL_02188 encoding an SsaA-like protein was highly overexpressed in *S. xylosus* (Table 1).

## 4. Conclusions

This study provides an extensive view of *S. xylosus* transcriptome when grown in a solid dairy matrix. Our transcriptome approach, combined with chemical analysis of the dairy matrix, has revealed some physiological adaptation to this peculiar medium, some of which significantly differ from lactic acid bacteria physiology in similar conditions. *S. xylosus* uses lactose and citrate as substrates but contrary to lactic acid bacteria, it adopts respiratory metabolism rather than fermentation. *S. xylosus* overcomes the amino acid deficiency of this environment by using urea as a source of nitrogen and overexpresses amino acid synthesis pathways. *S. xylosus* must cope with several stresses, some of which are regulated by the SigB regulator, and may use an original system for iron acquisition from lactoferrin. As *S. xylosus* is part of the microbial cheese ripening community, this study provides knowledge that can be used to analyze the transcriptomic data of this community of several species or to analyze the metatranscriptomic data of the whole microbial community of cheeses.

## Figures and Tables

**Table 1:** Genes of *Staphylococcus xylosus* differentially expressed at 24 h and/or 48 h compared to 6 h in the dairy matrix model.

Gene ID	Gene Name	Description	Mean Ratio of Expression	
			24 h/6 h	48 h/6 h
		**Cellular Processes**		
SXYL_02062	*rnr*	Ribonuclease R	2.7	2.1
SXYL_02088-89	*uvrAB*	UvrABC system protein A and B	3.1 *	2.5 *
SXYL_01472	*cspA*	Cold shock protein CspA	0.3	0.5
SXYL_01630-31	*hslUV*	ATP-dependent protease ATPase	2.4 *	2.1 *
**Carbohydrate Metabolism**				
**Transporters**				
SXYL_00773-76	*mtlDFA*	Mannitol transport system	9.9 *	7.1 *
SXYL_00268-70	*ulaA*	PTS ascorbate transporter	3.9 *	3.2
SXYL_00255-60		PTS sugar transport system	12.1 *	7.6 *
**Regulator**				
SXYL_01129	*ccpA*	Catabolite control protein A		2.1
**Galactose/lactose**				
SXYL_00082	*lacR*	Lactose operon transcription activator	0.5	0.5
SXYL_00672-74	*galKET*	Galactokinase, UDP-glucose 4-epimerase and galactose-1-phosphate uridylyltransferase	4.0 *	
SXYL_01351	*malA*	Alpha-D-1,4-glucosidase	2.3	2.2
**Pentose phosphate pathway/pentose and glucuronate interconversions**				
SXYL_01353	*zwf*	Glucose-6-phosphate 1-dehydrogenase	2.3	2.2
SXYL_02290		6-phospho-3-hexuloisomerase	13.7	9.3
SXYL_02291		3-hexulose-6-phosphate synthase	17.5	12.5
SXYL_00568	*rpi*	Ribose-5-phosphate isomerase	3.0	3.4
SXYL_02430	*prs*	Ribose-phosphate pyrophosphokinase	2.4	2.4
SXYL_02582		6-phosphogluconate dehydrogenase	2.9	4.2
SXYL_02337-39	*uxuAC2*	D-mannonate oxidoreductase, Mannonate dehydratase and Glucuronate isomerase	2.9 *	2.0 *
SXYL_00132	*xylE*	Xylose transporter		2.0
SXYL_00133-34	*xylBA*	D-xylulose kinase and xylose isomerase	3.7 *	4.5 *
SXYL_02250		Xylose isomerase	3.7	3.3
SXYL_00607	*araB1*	Ribulokinase	2.6	2.0
SXYL_02343		2-keto-3-deoxygluconate kinase	2.0	
**Glycolysis**				
SXYL_00221	*fda*	Fructose-bisphosphate aldolase class 1	2.2	2.7
SXYL_01169	*pfkA*	6-phosphofructokinase	2.7	
SXYL_02071	*gapR*	Glycolytic operon regulator	3.6	3.4
**Pyruvate metabolism**				
SXYL_00276	*ldhB*	L-lactate dehydrogenase	2.6	4.8
SXYL_00170	*lqo*	L-lactate-quinone oxidoreductase	2.8	2.2
SXYL_00366-67	*cidBC*	Holin-like protein CidB and pyruvate oxidase	12.0 *	8.2 *
SXYL_01023-24	*pflAB*	Formate acetyltransferase	3.0 *	
SXYL_00431	*budA*	Alpha-acetolactate decarboxylase	2.6	2.3
**TCA cycle and respiratory chain**				
SXYL_01592-93		2-oxoglutarate ferredoxin oxidoreductase	4.6 *	5.2 *
SXYL_00481		Citrate transporter	3.3	3.5
SXYL_02340-42		C4-dicarboxylate transport system	2.6 *	
SXYL_01012	*fumC*	Fumarate hydratase class II	2.2	2.7
SXYL_01172		Citrate synthase		2.1
SXYL_01173	*icd*	Isocitrate dehydrogenase [NADP]		2.1
SXYL_01821	*pyc*	Pyruvate carboxylase	0.5	0.3
SXYL_01534	*acnA*	Aconitate hydratase	3.1	2.5
SXYL_00218	*gabD*	Succinate-semialdehyde dehydrogenase	2.2	
SXYL_01849-50	*cydBA*	Cytochrome bd-type quinol oxidase	3.3 *	2.9 *
SXYL_00470	*ppk*	Polyphosphate kinase	6.7	4.9
SXYL_01851		Glutaredoxin	3.1	4.2
**Amino Acids Synthesis**				
**Transporters**				
SXYL_00265-66		ABC-type amino acid transport system		2.2 *
SXYL_00661		ABC-type amino acid transport system permease		2.0
SXYL_02264		Amino acid permease	6.5	8.3
SXYL_02687	*cstA*	Carbon starvation protein CstA	3.1	2.9
**Regulators**				
SXYL_01629	*codY*	GTP-sensing transcriptional pleiotropic repressor	2.2	3.1
**Histidine**				
SXYL_00460-64	*hisIFAHB*	Histidine biosynthesis	2.4 *	
SXYL_00465-68	*hisCDGZ*	Histidine biosynthesis	3.1 *	2.4 *
SXYL_02126	*hisC2*	Histidinol-phosphate aminotransferase	2.2	2.4
SXYL_00008	*hutH*	Histidine ammonia-lyase (Histidase)	2.0	
SXYL_00614	*hutG*	Formimidoylglutamase	7.6	7.7
SXYL_00617	*hutU*	Urocanate hydratase	2.2	
SXYL_00618	*hutI*	Imidazolonepropionase	3.8	3.7
**Glutamate, glutamine**				
SXYL_00106-08	*glnA2*	Short-chain dehydrogenase, glutamine synthetase and aldehyde dehydrogenase	25.5 *	15.1 *
SXYL_00476		Glutamate synthase	5.1	3.7
SXYL_02393	*gltX*	Glutamate--tRNA ligase	3.7	4.1
SXYL_00347	*rocA*	1-pyrroline-5-carboxylate dehydrogenase	2.4	2.2
SXYL_01964	*gluD1*	Glutamate dehydrogenase		2.1
**Arginine**				
SXYL_01961-62	*argGH*	Argininosuccinate synthase and lyase	3.0 *	6.1 *
SXYL_01965	*rocD2*	Ornithine aminotransferase 2	2.4	
SXYL_00252	*arcB*	Ornithine carbamoyltransferase		4.9
SXYL_00239-41	*argCJB*	Initial steps of the arginine biosynthetic pathway	3.5 *	4.4 *
SXYL_00769	*arg*	Arginase	2.3	
SXYL_01355	*proC*	Pyrroline-5-carboxylate reductase	3.2	4.6
**Urea catabolism**				
SXYL_00291-96	*ureGFECBA*	Urease	3.0 *	2.1 *
SXYL_00297		Urea transporter	2.2	2.0
**Lysine**				
SXYL_01476-79	*dapHBAasd*	Lysine biosynthesis	2.7 *	2.6 *
SXYL_01480		Aspartokinase	2.1	
SXYL_00377	*dapE*	Succinyl-diaminopimelate desuccinylase	2.0	
**Leucine, valine, isoleucine**				
SXYL_00867-69	*ilvAleuDCB*	Leucine, valine, isoleucine biosynthesis	4.7 *	2.9 *
SXYL_00870	*leuB*	3-isopropylmalate dehydrogenase	2.5	
SXYL_02469	*ilvD2*	Dihydroxy-acid dehydratase	3.0	3.0
**Glycine**				
SXYL_01317-19	*gcvTPAPB*	Glycine metabolism	4.7 *	3.1 *
**Tryptophan**				
SXYL_01497	*trpA*	Tryptophan synthase alpha chain		5.4
SXYL_01498-503	*trpBFCDGE*	Tryptophan biosynthesis	6.3 *	6.2 *
**Cysteine, methionine**				
SXYL_02630-38	*cysCsatcobAcysIJH*	Cysteine biosynthesis	41.6 *	39.0 *
SXYL_02417	*cysK*	Cysteine synthase	2.7	4.2
SXYL_02391-93	*cysSEgltX*	Cysteine--tRNA ligase, serine acetyltransferase and glutamate--tRNA ligase	4.5 *	3.8 *
SXYL_00283-85	*cysDMmetB*	Cysteine biosynthesis	5.9 *	6.2 *
SXYL_02641-42		Cystathionine gamma-synthase	23.0 *	23.1 *
SXYL_02643-45		Methionine biosynthesis	105.2 *	92.5 *
SXYL_01238		Cysteine desulfurase	4.3	3.2
SXYL_00118		Dihydrofolate reductase family protein	6.8	5.4
**Nucleic Acid Bases, Vitamins, Cofactors Syntheses**				
**Pyrimidine**				
SXYL_01686	*carB*	Carbamoyl-phosphate synthase large chain	2.2	
SXYL_01687-91	*carApyrCBPR*	UMP biosynthesis	7.2 *	6.6 *
SXYL_01249	*udk*	Uridine kinase		2.3
**Purine**				
SXYL_01861-64	*purDHNM*	IMP biosynthesis	3.0 *	
SXYL_01865-71	*purFLQCKE*	IMP biosynthesis	12.9 *	7.0 *
SXYL_00928	*purB*	Adenylosuccinate lyase (ASL)	3.0	3.4
**Thiamine**				
SXYL_02587	*thiC*	Phosphomethylpyrimidine synthase	2.1	
SXYL_00839	*thiD*	Hydroxy-phosphomethylpyrimidine kinase	2.4	
SXYL_00840	*thiM*	Hydroxyethylthiazole kinase	2.4	
**Panthotenate**				
SXYL_00168	*panB*	3-methyl-2-oxobutanoate hydroxymethyltransferase	3.2	2.4
SXYL_00169	*panC*	Pantothenate synthetase	3.1	2.3
SXYL_01178	*coaE*	Dephospho-CoA kinase	2.7	2.2
SXYL_00273		2-dehydropantoate 2-reductase	9.3	7.0
**Nicotinate**				
SXYL_00924		Nicotinate phosphoribosyltransferase	3.2	2.1
SXYL_00925	*nadE*	NH(3)-dependent NAD(+) synthetase	2.9	2.3
SXYL_01458		Dihydrofolate reductase family protein	2.0	
SXYL_02532	*nrlA*	NADPH-dependent oxidoreductase	18.9	13.1
**Lipids, Glycerolipids Metabolism**				
SXYL_02652		Acetyl-CoA acetyltransferase	4.9	2.7
SXYL_02653		NAD binding 3-hydroxyacyl-CoA dehydrogenase	4.7	2.1
SXYL_02654		Acyl-CoA dehydrogenase	2.8	
SXYL_01253-54		Acetyl-CoA carboxylase, biotin carboxyl carrier protein and biotin carboxylase	3.8 *	3.2 *
SXYL_00566-67	*dhaK2L2*	Dihydroxyacetone kinase, K and L subunits	4.5 *	4.0 *
SXYL_02198-200	*dhaMLK*	Dihydroxyacetone kinase, M, L and K subunits	3.5 *	2.5 *
SXYL_01576	*glpD*	Aerobic glycerol-3-phosphate dehydrogenase	2.2	2.5
SXYL_02208	*tagD*	Glycerol-3-phosphate cytidylyltransferase		2.1
SXYL_01997	*lipA*	Lipoyl synthase	2.2	2.0
SXYL_01915	*ugtP*	Processive diacylglycerol beta-glucosyltransferase	2.5	2.2
SXYL_01916	*ltaA*	Probable glycolipid permease LtaA	2.0	
		**Iron Transport**		
SXYL_00561-63		Oxidoreductase, monooxygenase and transporter	24.4 *	36.5 *
SXYL_00749	*sfaB*	Siderophore biosynthesis protein, IucA/IucC family	2.1	2.5
SXYL_00459		ABC-type cobalamin Fe3+-siderophores transporter	2.2	2.0
**Response to Stress**				
**General stress**				
SXYL_00859	*rsbU*	Serine phosphatase RsbU regulator of sigma subunit	0.4	0.3
SXYL_00860-62	*rsbVW, sigB*	Anti-sigma-B factor antagonist, Serine-protein kinase RsbW and RNA polymerase sigma factor	3.8 *	3.8 *
SXYL_00743	*opuD2*	Glycine betaine transporter	5.0	3.7
SXYL_00744	*amaP*	Alkaline shock response membrane anchor protein	13.2	11.3
SXYL_00745		DUF2273 domain-containing protein	11.6	11.2
SXYL_00746	*asp23*	Alkaline shock protein 23	9.2	12.8
SXYL_00309		Universal stress protein	2.2	2.2
SXYL_00196		General stress protein	11.0	8.6
SXYL_00548		Heat shock protein Hsp20		2.0
**Cation transport**				
SXYL_00416		Zinc ABC transporter substrate-binding protein	2.2	2.2
SXYL_00559		Na/Pi cotransporter family protein		2,3
SXYL_01831	*mntH*	Divalent metal cation transporter MntH	2.2	2.6
SXYL_01859		ABC-type cobalt transport system ATPase	2.4	
SXYL_02658-60	*mtsC*	Metal ABC transporter	3.9 *	4.4 *
SXYL_00784	*czrA*	Zinc and cobalt transport repressor CzrA	5.5	6.1
**Phosphate**				
SXYL_01484-87	*pstSCAB*	Phosphate transporter	2.5 *	3.3 *
SXYL_02189		Inorganic phosphate transporter		2.3
SXYL_00470-71	*ppk, ppx*	Polyphosphate kinase and Exopolyphosphatase	8.2 *	5.4 *
**Pigmentation**				
SXYL_00050	*crtO*	Glycosyl-4,4 -diaponeurosporenoate acyltransferase	4.7	2.8
SXYL_00051-53	*crtPQM*	Diapolycopene oxygenase, 4,4 -diaponeurosporenoate glycosyltransferase and dehydrosqualene synthase	2.5 *	
**Oxidative stress**				
SXYL_00923	*nos*	Nitric oxide synthase oxygenase	3.2	2.3
SXYL_02533	*katC*	Catalase C	20.8	12.9
SXYL_02505	*katA*	Catalase A	0.2	0.1
SXYL_01303	*sodA*	Superoxide dismutase [Mn/Fe]	2.8	4.1
SXYL_02639	*hmpA*	Flavohemoprotein	2.5	2.5
SXYL_02418	*hslO*	33 kDa chaperonin	2.5	2.2
**Osmotic stress**				
SXYL_02127		Glycine/betaine/choline ABC transporter	2.4	2.0
SXYL_02128		Glycine/betaine/choline ABC transporter ATPase	2.1	
SXYL_00486	*lcdH*	L-carnitine dehydrogenase	4.9	3.9
SXYL_00407		Sodium:solute symporter	3.0	
SXYL_00232		MFS transporter	2.6	3.1
SXYL_00286		MFS transporter	4.2	3.8
SXYL_00379-82	*capBCAE*	Polyglutamate synthesis	2.2 *	2.5 *
SXYL_00114-15	*saeRS*	Response regulator and Histidine protein kinase	0.4 *	0.2 *
SXYL_00323	*isaA*	Transglycosylase IsaA	3.7	5.1
SXYL_00116	*sceD2*	Transglycosylase SceD 2	20.2	27.8
SXYL_00117	*sceD1*	Transglycosylase SceD1	116.5	124.1
SXYL_02188		Secretory antigen SsaA-like protein	27.9	36.1

* Means of the expression of the clustered genes differentially expressed.

## Supplementary Materials

Supplementary materials can be found at https://www.mdpi.com/2076-2607/8/11/1807/s1, Table S1: Targeted genes of Staphylococcus xylosus for the validation of microarray data by qPCR: expression at T24 h and T48 h in solid dairy matrix compared with T6 h. Table S2: Total genes of *Staphylococcus xylosus* differentially expressed at 24 h and/or 48 h of incubation compared to 6 h in solid dairy matrix.

## References

[1] Dugat-Bony E., Garnier L., Denonfoux J., Ferreira S., Sarthou A.-S., Bonnarme P., Irlinger F. (2016). Highlighting the microbial diversity of 12 French cheese varieties. Int. J. Food Microbiol..

[2] Montel M.-C., Buchin S., Mallet A., Delbes-Paus C., Vuitton D.A., Desmasures N., Berthier F. (2014). Traditional cheeses: Rich and diverse microbiota with associated benefits. Int. J. Food Microbiol..

[3] Kergourlay G., Taminiau B., Daube G., Champomier-Vergès M.-C. (2015). Metagenomic insights into the dynamics of microbial communities in food. Int. J. Food Microbiol..

[4] Wolfe B.E., Button J.E., Santarelli M., Dutton R.J. (2014). Cheese Rind Communities Provide Tractable Systems for In Situ and In Vitro Studies of Microbial Diversity. Cell.

[5] Niccum B.A., Kastman E.K., Kfoury N., Robbat A., Wolfe B.E. (2020). Strain-Level Diversity Impacts Cheese Rind Microbiome Assembly and Function. mSystems.

[6] Oikonomou G., Addis M.F., Chassard C., Nader-Macias M.E.F., Grant I., Delbès C., Bogni C.I., Le Loir Y., Even S. (2020). Milk Microbiota: What Are We Exactly Talking About?. Front. Microbiol..

[7] Delbes C., Ali-Mandjee L., Montel M.-C. (2007). Monitoring Bacterial Communities in Raw Milk and Cheese by Culture-Dependent and -Independent 16S rRNA Gene-Based Analyses. Appl. Environ. Microbiol..

[8] Aldrete-Tapia A., Escobar-Ramírez M.C., Tamplin M.L., Hernández-Iturriaga M. (2014). High-throughput sequencing of microbial communities in Poro cheese, an artisanal Mexican cheese. Food Microbiol..

[9] Riquelme C., Câmara S., Dapkevicius M.D.L.N.E., Vinuesa P., Da Silva C.C.G., Malcata F.X., Rego O.A. (2015). Characterization of the bacterial biodiversity in Pico cheese (an artisanal Azorean food). Int. J. Food Microbiol..

[10] Ryssel M.B., Jespersen L., Abu Al-Soud W., Sørensen S., Arneborg N., Jespersen L. (2015). Microbial diversity and dynamics throughout manufacturing and ripening of surface ripened semi-hard Danish Danbo cheeses investigated by culture-independent techniques. Int. J. Food Microbiol..

[11] Larpin-Laborde S., Imran M., Bonaïti C., Bora N., Gelsomino R., Goerges S., Irlinger F., Goodfellow M., Ward A.C., Vancanneyt M. (2011). Surface microbial consortia from Livarot, a French smear-ripened cheese. Can. J. Microbiol..

[12] Addis E., Fleet G., Cox J., Kolak D., Leung T. (2001). The growth, properties and interactions of yeasts and bacteria associated with the maturation of Camembert and blue-veined cheeses. Int. J. Food Microbiol..

[13] Bockelmann W., Willems K., Neve H., Heller K. (2005). Cultures for the ripening of smear cheeses. Int. Dairy J..

[14] Cretenet M., Laroute V., Ulvé V., Jeanson S., Nouaille S., Even S., Piot M., Girbal L., Le Loir Y., Loubière P. (2010). Dynamic Analysis of the *Lactococcus lactis* Transcriptome in Cheeses Made from Milk Concentrated by Ultrafiltration Reveals Multiple Strategies of Adaptation to Stresses. Appl. Environ. Microbiol..

[15] Taïbi A., Dabour N., Lamoureux M., Roy D., Lapointe G. (2011). Comparative transcriptome analysis of *Lactococcus lactis* subsp. *cremoris* strains under conditions simulating Cheddar cheese manufacture. Int. J. Food Microbiol..

[16] De Filippis F., Genovese A., Ferranti P., Gilbert J.A., Ercolini D. (2016). Metatranscriptomics reveals temperature-driven functional changes in microbiome impacting cheese maturation rate. Sci. Rep..

[17] Porcellato D., Skeie S.B. (2016). Bacterial dynamics and functional analysis of microbial metagenomes during ripening of Dutch-type cheese. Int. Dairy J..

[18] Levante A., De Filippis F., La Storia A., Gatti M., Neviani E., Ercolini D., Lazzi C. (2017). Metabolic gene-targeted monitoring of non-starter lactic acid bacteria during cheese ripening. Int. J. Food Microbiol..

[19] Duru I.C., Laine P.K., Andreevskaya M., Paulin L., Kananen S., Tynkkynen S., Auvinen P., Smolander O.-P. (2018). Metagenomic and metatranscriptomic analysis of the microbial community in Swiss-type Maasdam cheese during ripening. Int. J. Food Microbiol..

[20] Arfi K., Leclercq-Perlat M.-N., Baucher A., Tache R., Delettre J., Bonnarme P. (2004). Contribution of several cheese-ripening microbial associations to aroma compound production. Le Lait.

[21] Sørensen L.M., Gori K., Petersen M.A., Jespersen L., Arneborg N. (2011). Flavour compound production by *Yarrowia lipolytica*, *Saccharomyces cerevisiae* and *Debaryomyces hansenii* in a cheese-surface model. Int. Dairy J..

[22] Monnet C., Dugat-Bony E., Swennen D., Beckerich J.-M., Irlinger F., Fraud S., Bonnarme P. (2016). Investigation of the Activity of the Microorganisms in a Reblochon-Style Cheese by Metatranscriptomic Analysis. Front. Microbiol..

[23] Pham N.-P., Landaud S., Lieben P., Bonnarme P., Monnet C. (2019). Transcription Profiling Reveals Cooperative Metabolic Interactions in a Microbial Cheese-Ripening Community Composed of *Debaryomyces hansenii*, *Brevibacterium aurantiacum*, and *Hafnia alvei*. Front. Microbiol..

[24] Mansour S., Bailly J., Landaud S., Monnet C., Sarthou A.S., Cocaign-Bousquet M., Leroy S., Irlinger F., Bonnarme P. (2009). Investigation of Associations of *Yarrowia lipolytica*, *Staphylococcus xylosus*, and *Lactococcus lactis* in Culture as a First Step in Microbial Interaction Analysis. Appl. Environ. Microbiol..

[25] Cretenet M., Nouaille S., Thouin J., Rault L., Stenz L., François P., Hennekinne J.-A., Piot M., Maillard M.B., Fauquant J. (2011). *Staphylococcus aureus* virulence and metabolism are dramatically affected by *Lactococcus lactis* in cheese matrix. Environ. Microbiol. Rep..

[26] Zdenkova K., Alibayov B., Karamonova L., Purkrtova S., Karpiskova R., Demnerova K. (2016). Transcriptomic and metabolic responses of *Staphylococcus aureus* in mixed culture with *Lactobacillus plantarum*, *Streptococcus thermophilus* and *Enterococcus durans* in milk. J. Ind. Microbiol. Biotechnol..

[27] Viçosa G.N., Botta C., Ferrocino I., Bertolino M., Ventura M., Nero L.A., Cocolin L. (2018). *Staphylococcus aureus* undergoes major transcriptional reorganization during growth with *Enterococcus faecalis* in milk. Food Microbiol..

[28] Ulve V., Monnet C., Valence-Bertel F., Fauquant J., Falentin H., Lortal S. (2008). RNA extraction from cheese for analysis of in situ gene expression of *Lactococcus lactis*. J. Appl. Microbiol..

[29] Evermassen A., La Foye A.E., Eloux V., Talon R., Leroy S. (2014). Transcriptomic analysis of *Staphylococcus xylosus* in the presence of nitrate and nitrite in meat reveals its response to nitrosative stress. Front. Microbiol..

[30] Smyth G.K. (2004). Linear Models and Empirical Bayes Methods for Assessing Differential Expression in Microarray Experiments. Stat. Appl. Genet. Mol. Biol..

[31] Livak K.J., Schmittgen T.D. (2001). Analysis of relative gene expression data using real-time quantitative PCR and the 2-ΔΔCT Method. Methods.

[32] Vermassen A., Dordet-Frisoni E., De La Foye A., Micheau P., Laroute V., Leroy S., Talon R. (2016). Adaptation of *Staphylococcus xylosus* to Nutrients and Osmotic Stress in a Salted Meat Model. Front. Microbiol..

[33] Turgay K. (2010). Role of Proteolysis and Chaperones in Stress Response and Regulation. Bacterial Stress Responses.

[34] Brückner R., Rosenstein R., Fischetti V., Novick R., Ferretti J., Portnoy D., Rood J. (2006). Carbohydrate catabolism: Pathways and Regulation. Gram-Positive Pathogens.

[35] Leroy S., Vermassen A., Ras G., Talon R. (2017). Insight into the Genome of *Staphylococcus xylosus*, a Ubiquitous Species Well Adapted to Meat Products. Microorganisms.

[36] Purves J., Cockayne A., Moody P.C.E., Morrissey J.A. (2010). Comparison of the Regulation, Metabolic Functions, and Roles in Virulence of the Glyceraldehyde-3-Phosphate Dehydrogenase Homologues *gapA* and gapB in *Staphylococcus aureus*. Infect. Immun..

[37] Chaudhari S.S., Thomas V.C., Sadykov M.R., Bose J.L., Ahn D.J., Zimmerman M.C., Bayles K.W. (2016). The LysR-type transcriptional regulator, CidR, regulates stationary phase cell death in *Staphylococcus aureus*. Mol. Microbiol..

[38] Thomas V.C., Sadykov M.R., Chaudhari S.S., Jones J., Endres J.L., Widhelm T.J., Ahn J.-S., Jawa R.S., Zimmerman M.C., Bayles K.W. (2014). A Central Role for Carbon-Overflow Pathways in the Modulation of Bacterial Cell Death. PLoS Pathog..

[39] Derzelle S., Bolotin A., Mistou M.-Y., Rul F. (2005). Proteome Analysis of *Streptococcus thermophilus* Grown in Milk Reveals Pyruvate Formate-Lyase as the Major Upregulated Protein. Appl. Environ. Microbiol..

[40] Raynaud S., Perrin R., Cocaign-Bousquet M., Loubière P. (2005). Metabolic and Transcriptomic Adaptation of *Lactococcus lactis* subsp. *lactis* Biovar diacetylactis in Response to Autoacidification and Temperature Downshift in Skim Milk. Appl. Environ. Microbiol..

[41] Dubey A.K., Baker C.S., Suzuki K., Jones A.D., Pandit P., Romeo T., Babitzke P. (2003). CsrA Regulates Translation of the *Escherichia coli* Carbon Starvation Gene, *cstA*, by Blocking Ribosome Access to the *cstA* Transcript. J. Bacteriol..

[42] Rasmussen J.J., Vegge C.S., Frøkiær H., Howlett R.M., Krogfelt K.A., Kelly D.J., Ingmer H. (2013). *Campylobacter jejuni* carbon starvation protein A (CstA) is involved in peptide utilization, motility and agglutination, and has a role in stimulation of dendritic cells. J. Med. Microbiol..

[43] Fiegler H., Brückner R. (2006). Identification of the serine acetyltransferase gene of *Staphylococcus xylosus*. FEMS Microbiol. Lett..

[44] Majerczyk C.D., Dunman P.M., Luong T.T., Lee C.Y., Sadykov M.R., Somerville G.A., Bodi K., Sonenshein A.L. (2010). Direct Targets of CodY in *Staphylococcus aureus*. J. Bacteriol..

[45] Nuxoll A.S., Halouska S.M., Sadykov M.R., Hanke M.L., Bayles K.W., Kielian T., Powers R., Fey P.D. (2012). CcpA Regulates Arginine Biosynthesis in *Staphylococcus aureus* through Repression of Proline Catabolism. PLoS Pathog..

[46] Schmid R., Uhlemann E.-M., Nolden L., Wersch G., Hecker R., Hermann T., Marx A., Burkovski A. (2000). Response to nitrogen starvation in *Corynebacterium glutamicum*. FEMS Microbiol. Lett..

[47] Brandenburg J.L., Wray J.L.V., Beier L., Jarmer H., Saxild H.H., Fisher S.H. (2002). Roles of PucR, GlnR, and TnrA in Regulating Expression of the *Bacillus subtilis* ure P3 Promoter. J. Bacteriol..

[48] Grundy F.J., Henkin T.M., Sonenshein A.L., Hoch J.A., Losick R. (2002). Synthesis of Serine, Glycine, Cysteine and Methionine. Bacillus subtilis and Its Closest Relatives: From Genes to Cells.

[49] Gollnick P., Babitzke P., Merino E., Yanofsky C., Sonenshein A.L., Hoch J.A., Losick R. (2002). Aromatic Amino Acid Metabolism in *Bacillus subtilis*. Bacillus subtilis and Its Closest Relatives: From Genes to Cells.

[50] Switzer R.L., Zalkin H., Saxild H.H., Sonenshein A.L., Hoch J.A., Losick R. (2002). Purine, Pyrimidine, and Pyridine Nucleotide Metabolism. Bacillus subtilis and Its Closest Relatives: From Genes to Cells.

[51] Monnet C., Back A., Irlinger F. (2012). Growth of Aerobic Ripening Bacteria at the Cheese Surface Is Limited by the Availability of Iron. Appl. Environ. Microbiol..

[52] Oda H., Wakabayashi H., Yamauchi K., Abe F. (2014). Lactoferrin and bifidobacteria. BioMetals.

[53] Jenssen H., Hancock R.E. (2009). Antimicrobial properties of lactoferrin. Biochimie.

[54] Duhutrel P., Bordat C., Wu T.-D., Zagorec M., Guerquin-Kern J.-L., Champomier-Vergès M.-C. (2009). Iron Sources Used by the Nonpathogenic Lactic Acid Bacterium *Lactobacillus sakei* as Revealed by Electron Energy Loss Spectroscopy and Secondary-Ion Mass Spectrometry. Appl. Environ. Microbiol..

[55] Vella G.S., Reznikov E.A., Monaco M., Donovan S. (2015). Regulation cell growth and virulence gene expression in *Staphylococcus aureus* by the iron-binding proteins lactoferrin and hemin. i-ACES.

[56] Vermassen A., Talon R., Leroy S. (2016). Ferritin, an iron source in meat for Staphylococcus xylosus?. Int. J. Food Microbiol..

[57] Pané-Farré J., Jonas B., Förstner K., Engelmann S., Hecker M. (2006). The σB regulon in *Staphylococcus aureus* and its regulation. Int. J. Med Microbiol..

[58] Bischoff M., Dunman P., Kormanec J., Macapagal D., Murphy E., Mounts W., Berger-Bächi B., Projan S. (2004). Microarray-based analysis of the *Staphylococcus aureus* Sigma B regulon. J. Bact..

[59] Stenz L., François P., Fischer A., Huyghe A., Tangomo M., Hernandez D., Cassat J., Linder P., Schrenzel J. (2008). Impact of oleic acid (cis-9-octadecenoic acid) on bacterial viability and biofilm production in *Staphylococcus aureus*. FEMS Microbiol. Lett..

[60] Müller M., Reiß S., Schlüter R., Mäder U., Beyer A., Reiß W., Marles-Wright J., Lewis R.J., Pförtner H., Völker U. (2014). Deletion of membrane-associated Asp23 leads to upregulation of cell wall stress genes in Staphylococcus aureus. Mol. Microbiol..

[61] Völker U., Engelmann S., Maul B., Riethdorf S., Völker A., Schmid R., Mach H., Hecker M. (1994). Analysis of the induction of general stress proteins of *Bacillus subtilis*. Microbiology.

[62] Nielsen E.W., Hui Y.H., Meunier-Goddik L., Hansen Å.S., Josephsen J., Nip W.-K., Stanfield P.S., Toldrà F. (2004). Principles of Cheese Production. Handbook of Food and Beverage Fermentation Technology.

[63] Bolesch D.G., Keasling J.D. (2000). Polyphosphate Binding and Chain Length Recognition of *Escherichia coli* Exopolyphosphatase. J. Biol. Chem..

[64] Rangarajan E.S., Nadeau G., Li Y., Wagner J., Hung M.-N., Schrag J.D., Cygler M., Matte A. (2006). The Structure of the Exopolyphosphatase (PPX) from *Escherichia coli* O157:H7 Suggests a Binding Mode for Long Polyphosphate Chains. J. Mol. Biol..

[65] Clauditz A., Resch A., Wieland K.-P., Peschel A., Götz F. (2006). Staphyloxanthin Plays a Role in the Fitness of *Staphylococcus aureus* and Its Ability to Cope with Oxidative Stress. Infect. Immun..

[66] Ras G., Zuliani V., Derkx P., Seibert T.M., Leroy S., Talon R. (2017). Evidence for Nitric Oxide Synthase Activity in *Staphylococcus xylosus* Mediating Nitrosoheme Formation. Front. Microbiology.

[67] Gusarov I., Nudler E. (2005). NO-mediated cytoprotection: Instant adaptation to oxidative stress in bacteria. Proc. Natl. Acad. Sci. USA.

[68] Vaish M., Singh V.K. (2013). Antioxidant Functions of Nitric Oxide Synthase in a Methicillin Sensitive *Staphylococcus aureus*. Int. J. Microbiol..

[69] Ras G., Leroy S., Talon R. (2018). Nitric oxide synthase: What is its potential role in the physiology of staphylococci in meat products?. Int. J. Food Microbiol..

[70] Reizer J., Reizer A., Saier M.H. (1994). A functional superfamily of sodium/solute symporters. Biochim. Et Biophys. Acta (BBA)-Rev. Biomembr..

[71] Pao S.S., Paulsen I.T., Saier M.H. (1998). Major Facilitator Superfamily. Microbiol. Mol. Biol. Rev..

[72] Kocianova S., Vuong C., Yao Y., Voyich J.M., Fischer E.R., DeLeo F.R., Otto M. (2005). Key role of poly-γ-dl-glutamic acid in immune evasion and virulence of *Staphylococcus epidermidis*. J. Clin. Investig..

[73] Stapleton M.R., Horsburgh M.J., Hayhurst E.J., Wright L., Jonsson I.-M., Tarkowski A., Kokai-Kun J.F., Mond J.J., Foster S.J. (2007). Characterization of IsaA and SceD, Two Putative Lytic Transglycosylases of *Staphylococcus aureus*. J. Bacteriol..

