# Supplementary material for: Transcriptomic Analysis of Staphylococcus xylosus in Solid Dairy Matrix Reveals an Aerobic Lifestyle Adapted to Rind
Source: Microorganisms. 2020 Nov 17;8(11):1807. doi: 10.3390/microorganisms8111807 (PMC7698506; doi:10.3390/microorganisms8111807)
Supplement: Supplementary file 1 [file microorganisms-08-01807-s001.zip › Supplementary Table S1.pdf]

Supplementary Table S1. Targeted genes of *Staphylococcus xylosus* for the validation of microarray data by qPCR: expression at T24h and T48h in solid dairy matrix compared with T6h.

Gene Id	Gene name	Sequences of primers pairs for qPCR (5'-3')		Mean ratio of expression			
				Microarray		qPCR	
		Forward	Reverse	24h	48h	24h	48h
SXYL_00370		ATGCCAAAAGTATCCTTAGTACCA	GCACGTTCATCTTCAATCGTC	0.2	0.2	0.1	0.1
SXYL_02535	*ahpF*	AACGCACACCAAGTTTCTCA	TTAATGCTTGAACAACGTCAGG	0.3	0.3	0.1	0.0
SXYL_00197		ATAGTCGTGGCTTTCTTCCTAC	ATTGAACTTGAGATACATTATCAGGC	15.7	13.6	8.2	7.0
SXYL_01277	*dnaK*	TTAGGTACAACAAATTCATGTATTGCA	GATAGATTGGATTGTATTAGGGTTAGTGA	0.4	0.4	0.1	0.1
SXYL_02088	*uvrA*	AGCGTAGATATGTTGAATCATTAAGTG	GGTTGCAACAGTAGATCTTGGA	2.3	2.1	2.4	2.2
SXYL_02230		GAAACAACTTTCCATAATTCGTCTTT	ATCATATACTTCAACAACTTGAGCT	0.5	0.4	0.2	0.1
SXYL_02534	*ahpC*	CACAGCTAACGCTTATGATCC	CGAATGAGAAGTCTGCTGGG	0.3	0.3	0.1	0.1
SXYL_00173		GGAAGTTGGATAGAGCCACAA	TTCTACACGATCCGCAATATCTAAT	12.2	6.8	11.4	5.7
SXYL_00607	*araB1*	ATGGGCACAGTGTCAATAGT	TTACGAGGTAATCGCCTGC	2.6	2.0	2.2	2.1
SXYL_01961	*argG*	TGGTTTAGATACAAGTGTCGCA	TGCATATGACACGAAATCTTCAC	3.0	6.9	2.7	6.3
SXYL_00226	*betA*	TTCAGTACTAGGTGGCCGAT	AAACTTTACCACGTGCATGTC	1.6	1.6	0.9	1.2
SXYL_00108		ATGGTGAGCAAATTCAAACTGAAA	AAGCATTAGCAGCGCTATCAA	21.6	14.1	17.1	7.6
SXYL_02630	*cysC*	GCATGCGTCAGAAGTAACTAAAG	CGATCTTCTGGACTAAATCCAAGAT	25.1	29.9	17.5	37.4
SXYL_02638	*cysH*	GTACGAAACATATGGCGATTCTATTAT	TAACAACATTGATTCGGATCGTTC	20.0	18.5	19.3	23.1
SXYL_02636	*cysI*	ACAGACAGTGATTATCTTCGTGG	TATGTGTTTGCGATATCGTCCAT	49.2	43.5	46.9	54.0
SXYL_00784	*czrA*	GGAAGATTCATTTAACGAACAAACG	TTGGTGAGAAACGTTAGATTGACT	5.5	6.1	4.1	4.5
SXYL_02149	*fruK*	AGGAGGCTTTCCAGGACAA	GTGGACCAGGCGCATTAAT	0.1	0.4	0.1	0.2
SXYL_00101		GCAGAAGAAGGTGCAAACG	GCGTTATTGACCAAGCCATC	2.3	2.1	2.2	2.0
SXYL_00107	*glnA2*	GGTAAGCGAATTACTGGAGACTT	CCGTCTACGGAGTAAACATCA	25.5	14.6	37.2	14.9
SXYL_02582		ACGCCTGTAGCTCAAATAGTG	GGCTGCGAATTCTCTGGAAT	2.9	4.2	2.1	3.8
SXYL_00467	*hisG*	GCGTTAGAACAGAGAGAACGT	GCCAAATGGTAAGTCCAGAAG	3.4	2.6	4.3	2.9
SXYL_00618	*hutI*	GATGGCAAAGTAGTTTATGCAGG	TCCACCTTGTTCCAGAATTTCTA	3.8	3.7	3.1	2.9
SXYL_00276	*ldhB*	GGAAGAGTAGGTAGTCAAGTTTTAAC	CTAATAATGTACGATCGCCATCATT	2.6	4.8	3.2	5.0
SXYL_00868	*leuD*	CAGGTAAAGTAGTGCCATTATTCC	ACCAGTAATTAAAATTGAAGCACCTT	5.0	3.0	4.7	2.4
SXYL_01974	*mnhE1*	CGTCACAGGCAGTTATACATTTAATAA	CGGCTCATTATCAATCTTTGGTTT	0.3	0.3	0.1	0.1
SXYL_02147	*nagA*	GGACGCTTCATTTGAAGGATT	TCCCAACAATACTAGCTGCAT	0.5	0.5	0.3	0.2
SXYL_01002		CATAGAGCGAGAGTGACGG	ACCACTACCTCGTTCACCTT	0.3	0.3	0.1	0.1
SXYL_01803	*pheT*	TAACGTGCTTGCTGAACGTAT	TGGCTCTACTTCACCAATGTC	0.4	0.5	0.3	0.6
SXYL_01869	*purC*	GTGGTAAGAAATATTGCTGCAGG	GAAGTCTACTAACCTTAAGTTCATTTCA	15.5	8.0	8.6	3.4
SXYL_01870	*purK*	CAACAATCGGCATTATTGGTGG	ACCAAGTTGTTCCAATGCTTC	19.1	10.8	10.0	5.7
SXYL_02631	*sat*	AACTCGATGAAATGGAACGC	GGCGCACGTACTCTAATCATAA	47.8	47.2	76.9	83.4
SXYL_00837		AACAGTAATCGCATCAACTTTAGC	CCTTCTTGAACTGGTGCTTCA	125.1	125.6	121.3	108.8
SXYL_00841	*thiE*	GTGGTACTCAAGATATACCAGTATCG	GGTACATTATAAGCGTGACAAAGC	1.9	1.8	1.2	1.7
SXYL_01498	*trpB*	GATTATGTCGGCCGTGAAAC	CGTTTAGCCAACAGTGCTTG	7.9	8.2	10.0	19.3
SXYL_01500	*trpC*	TGCTCGAACAGGGCTATTATG	GTGCTAAATCTCTACCTGGCA	7.0	7.1	8.0	12.2
SXYL_00296	*ureA*	GCAGCAGATTTAGCTCGTAGA	CCTCCATTACATCATCCTCACC	2.8	2.0	2.7	2.1
SXYL_00417		GTAAATCAACAGACACTATGGACG	GGGTATTAATAACTTAACGTGATCTGAG	25.0	18.6	19.1	20.7
SXYL_02348		TCAACACTTGTAGTAGGTTCTTCAG	TTCAGCTTTTAGTTCTGCTTTACTTT	109.8	124.6	121.7	191.1
SXYL_02642		GAAGTAATATTCGATGCACATAGAGG	ACCTTCCAGTTTAGCTAGCTTT	28.7	28.4	15.5	23.2
SXYL_02634		GTCTTATATGTAAGTCACGGTAGTAGAG	GTTCAATTTCTGATGGAATGTCCTT	46.8	45.4	49.1	79.9
SXYL_02644	*metE*	TTACACAAAGAGAACTTATTACTCCAAA	ATATAGTGATAGTTTGTGTTGAACCATT	124.1	115.3	90.9	105.2
SXYL_02645		ATTAGAACAATTAAAATCATCAACGTGG	AGTACCATACTGACTTACAATATTCCAA	112.5	99.0	107.4	107.6
SXYL_00255		GACGTATGAAAATGGATAAGAATTTGATG	TCATCAGCAAAATAAGGAAATGCTTT	15.0	9.0	12.6	8.3
SXYL_00744	*amaP*	GGCATATTAATTGTAGTAATCGTTGGA	GACCAATAATAATGAGTAACGCTGC	13.2	11.3	9.3	10.1
